# Aging‐related metabolic changes in the extensor digitorum longus muscle of senescence‐accelerated mouse‐prone 8

**DOI:** 10.1111/ggi.14333

**Published:** 2021-12-22

**Authors:** Teruhide Hoshino, Yoshiaki Kato, Keisuke Sugahara, Akira Katakura

**Affiliations:** ^1^ Department of Oral Pathobiological Science and Surgery Tokyo Dental College Tokyo Japan

**Keywords:** digitorum longus muscle, metabolome analysis, sarcopenia, senescence‐accelerated mouse‐prone 8

## Abstract

**Aim:**

Sarcopenia – aging‐related loss of muscle mass and muscle strength – is a key feature of the frailty model. In the present study, we aimed to elucidate the molecular biological changes associated with aging in the extensor digitorum longus muscle of senescence‐accelerated mouse prone 8 mouse model by capillary electrophoresis–mass spectrometry.

**Methods:**

Three groups of senescence‐accelerated mouse prone 8 mice were used, namely, 12‐week‐old (young; *n* = 5), 40‐week‐old (elderly; *n* = 5) and 55‐week‐old mice (late elderly; *n* = 5). The extensor digitorum longus muscle was collected. After preliminary analyses, metabolome analysis was carried out by capillary electrophoresis–mass spectrometry. Additionally, we examined whether the activity of enzymes in the metabolic pathway fluctuated with aging, by real‐time polymerase chain reaction.

**Results:**

Among the 116 water‐soluble metabolites associated with the central energy metabolism pathway, changes were observed in 19 metabolites between 12‐ and 40 ‐weeks‐old, in 40 metabolites between 40‐ and 55‐weeks‐old, and in 57 metabolites between 12‐ and 55‐weeks‐old. The fluctuated metabolites that were common among the groups were Val, putrescine and His. The levels of putrescine, associated with cell proliferation, protein synthesis and nucleic acid synthesis, and β‐Ala and His, a component of carnosine that is characterized by its anti‐oxidant and anti‐fatigue effects, decreased with age.

**Conclusions:**

We confirmed that there were two aging‐related metabolic changes in the extensor digitorum longus muscle of senescence‐accelerated mouse prone 8 mice. Based on the changes in metabolites, cell senescence and fatigue in the extensor digitorum longus muscle might increase in old mice compared with those in young mice, showing molecular biological changes with aging. **Geriatr Gerontol Int 2022; 22: 160–167**.

## Introduction

Sarcopenia – aging‐related loss of muscle mass and strength – is a key feature of the frailty model proposed by Fried *et al*.^1^ Humans aged ≥30 years lose muscle mass at a rate of approximately 5% per decade; individuals aged ≥60 years lose muscle mass at a faster rate.^2^ Sarcopenia is characterized by atrophy of fast‐twitch muscle selectively, and thus, early detection and prevention of sarcopenia and frailty are important.^3^

To date, several studies have observed aging‐related morphological and molecular biological changes in muscles. Senescence‐accelerated mouse‐prone 8 (SAMP8) has been commonly used in aging studies. Guo *et al*. observed aging‐related morphological and immunohistochemical changes in the gastrocnemius muscle of SAMP8 mice.^4^ Derave *et al*. reported that the wet weight of soleus muscle in SAMP8 mice decreases with age.^5^ We observed chronological changes characterized by muscular atrophy and contraction in masseter muscle of aged SAMP8 mice compared with those in young mice.^6^ However, there are a few reports of molecular biological changes associated with aging. Studies have focused on the metabolites of muscle‐related aging. In the present study, we aimed to identify metabolites related to aging in the lower limbs of SAMP8 mice, by elucidating the metabolic changes associated with aging in the extensor digitorum longus muscle (EDL), by capillary electrophoresis–mass spectrometry (CE‐MS). By clarifying the specific metabolic changes, the present study might contribute to the prevention and early detection of frailty.

## Methods

### Experimental animals

Male SAMP8 mice (SAMP8/Ta Slc) were purchased from Sankyo Labo Service Corporation (Tokyo, Japan). Three groups of male SAMP8 mice (SAMP8/TaSlc) were used in the present study, namely, 12‐week‐old (young; *n* = 5), 40‐week‐old (elderly; *n* = 5) and 55‐week‐old mice (late elderly; *n* = 5).

We examined the mice at the age of 12 weeks (accelerated senescence period), 40 weeks (mean lifespan) and 55 weeks (progressing age period). The mice were raised in a 125 × 213 × 125 mm aluminum cage, and were provided feed (Lab MR‐A1) and water ad libitum. The mice were anesthetized with isoflurane and euthanized by dislocating the cervical spines; thereafter, the EDL was extracted immediately. The left side of the muscle was used for metabolome analysis and the right side was used for real‐time polymerase chain reaction. The present study was carried out according to the Animal Research Ethics Committee Guidelines at Tokyo Dental College (approval number: 202601).

### Quantitative analysis of myosin heavy chain isoform

For gene expression analysis, the TaqMan gene expression assay kit (Thermo Fisher Scientific, Waltham, MA, USA) and the RT–PCR 7500 real‐time polymerase chain reaction system (Thermo Fisher Scientific) were used. The total RNA from the EDL was extracted using the RNeasy Mini Kit (Qiagen, Hilden, Germany) and Proteinase K (Takara Bio, Shiga, Japan). TaqMan probes (Thermo Fisher Scientific) for each isoform of the myosin heavy chain (MyHC) was as follows: *MyHC‐1* (Mm01319006.g1), *MyHC‐2b* (Mm01332518.m1), *MyHC‐2a* (Mm01332564.m1) and *MyHC‐2x/d* (Mm01332489.m1). In addition, *β‐actin* (Mm00607939_s1) was used as a housekeeping gene. The cDNA was prepared using the QuantiTect Reverse Transcription Kit (Thermo Fisher Scientific). The Double Delta Ct Value (ΔΔCt) method was used for quantification, and the relative expression level was compared with the expression of housekeeping genes in each sample.

### Metabolite extraction

Metabolome analysis was carried out at Human Metabolome Technologies (Tsuruoka, Japan). Approximately 10–20 mg of frozen EDL sample was plunged in 225–750 μL of 50% acetonitrile/milli‐Q water containing internal standards (H3304‐1002, Human Metabolome Technologies) at 0°C to inactivate enzymes. The tissue was homogenized three times at 3500 rpm for 3 min using a tissue homogenizer (Micro Smash MS100R; Tomy Digital Biology, Tokyo, Japan), and then the homogenate was centrifuged at 2300 *g* for 5 min at 4°C. Subsequently, 400 μL of the upper aqueous layer was filtered by centrifugation through a Millipore 5 kDa cut‐off filter at 9100 *g* for 120 min at 4°C to remove proteins. The filtrate was concentrated by centrifugation and re‐suspended in 50 μL of milli‐Q water for capillary electrophoresis–mass spectrometry analysis.

### Metabolome analysis

Metabolome analysis was carried out by capillary electrophoresis time‐of‐flight mass spectrometry for cations and capillary electrophoresis–tandem mass spectrometry for anions, as described previously.^7^, ^8^ Briefly, capillary electrophoresis time‐of‐flight mass spectrometry analysis was carried out using an Agilent CE capillary electrophoresis system equipped with an Agilent 6210 time‐of‐flight mass spectrometer (Agilent Technologies, Waldbronn, Germany). The systems were controlled by Agilent G2201AA ChemStation software version B.03.01 for CE (Agilent Technologies) and connected to a fused silica capillary (50 μm i.d. × 80 cm total length) with commercial electrophoresis buffer (H3301‐1001 and I3302‐1023 for cation and anion analyses, respectively; Human Metabolome Technologies) as the electrolyte. The spectrometer was scanned from an *m/z* ratio of 50 to 1000.^7^ Peaks were extracted using MasterHands, automatic integration software (Keio University, Tsuruoka, Yamagata, Japan), and MassHunter Quantitative Analysis B.04.00 (Agilent Technologies) to obtain the *m/z* value, peak area and migration time. Signal peaks were annotated according to the Human Metabolome Technologies metabolite database based on their *m*/*z* values with the migration times. The concentrations of metabolites were calculated by normalizing the peak area of each metabolite with respect to the area of the internal standard and by using standard curves with three‐point calibrations. Hierarchical cluster analysis and principal component analysis (PCA) were carried out using proprietary software, PeakStat and SampleStat, respectively.

### Quantitative analysis of expression in metabolites fluctuated with aging

We focused on the polyamine metabolic pathways, because clear metabolic fluctuations were observed between 40‐ and 55‐week‐old mice. For target genes in the polyamine metabolic pathway, the TaqMan probes (Thermo Fisher Scientific) used were methionine adenosyl transferase 2A (*Mat2a*; Mm00728688_s1), spermidine synthase (*Srm*; Mm00726089_s1), spermine synthase (*Sms*; Mm00786246_s1), ornithine decarboxylase1 (*Odc1*; Mm02019269_g1), spermine oxidase (*Smox*; Mm01275475_m1) and S‐adenosylmethionine decarboxylase (*Amd2*; Mm04207265_gH).

### Statistical analysis

Tukey's test was used for comparison of MyHC isoform among the three groups. Furthermore, Welch's *t*‐test was used for the comparison of metabolites between two groups. The significance threshold was 5% (*P* < 0.05).

## Results

### Quantitative analysis of MyHC isoform

**Figure 1:**
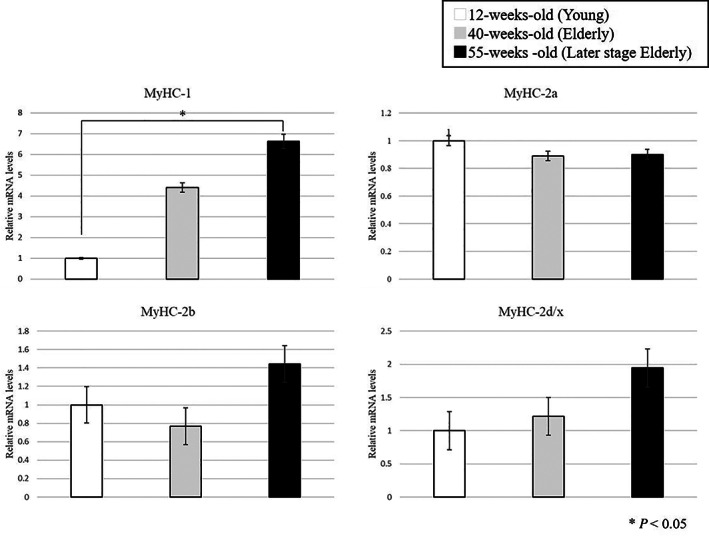
Level of *MyHC‐1*, *MyHC‐2a*, *MyHC‐2b* and *MyHC‐2d/x* mRNA expression. Expression of *MyHC‐1* increased between 12 and 55 weeks‐of‐age. There was a significant difference (*P* < 0.05).

The composition of type fibers in MyHC changed with aging (Fig. 1). The expression of *MyHC‐1* and *MyHC‐2x/d* tended to increase with aging. Furthermore, the expression of *MyHC‐2a* and *MyHC‐2b* tended to decrease between 12‐ and 40‐weeks‐old. The expression of *MyHC‐1* increased between 12‐ and 55‐weeks‐old, and there was a significant difference (*P* < 0.05).

#### Metabolome analysis

**Figure 2:**
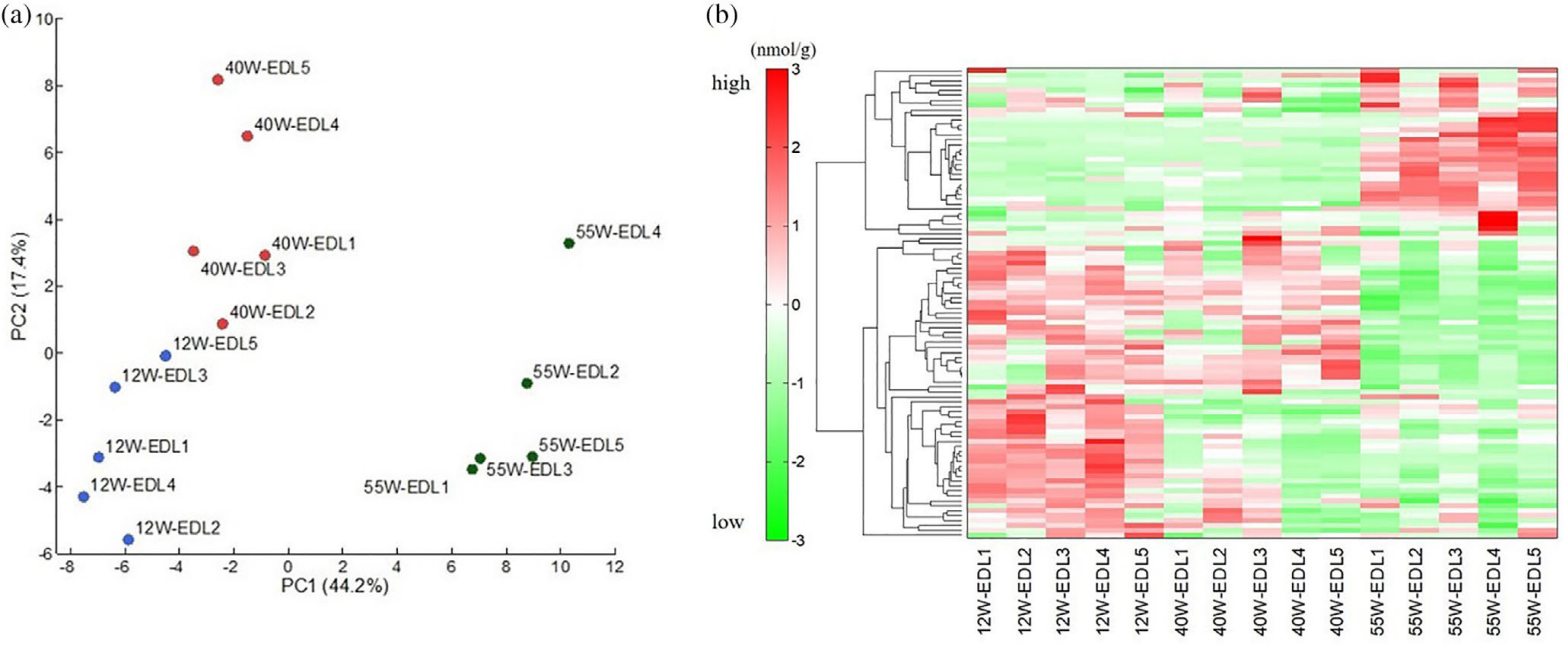
(a) Principal component analysis of metabolomic datasets of skeletal muscle from extensor digitorum longus muscle (EDL). The principal component analysis was carried out with the peak data using SampleStat version 3.14. Plots of young (blue circles), elderly (red circles) and late elderly (green circles) are clearly distinguished on the first principal component axis (*x*‐axis). (b) Heat map comparing metabolite changes. The heat map patterns were clearly distinguishable. Red indicates that the relative content of metabolites is high, whereas green indicates that the relative content of metabolites is low.

**Figure 3:**
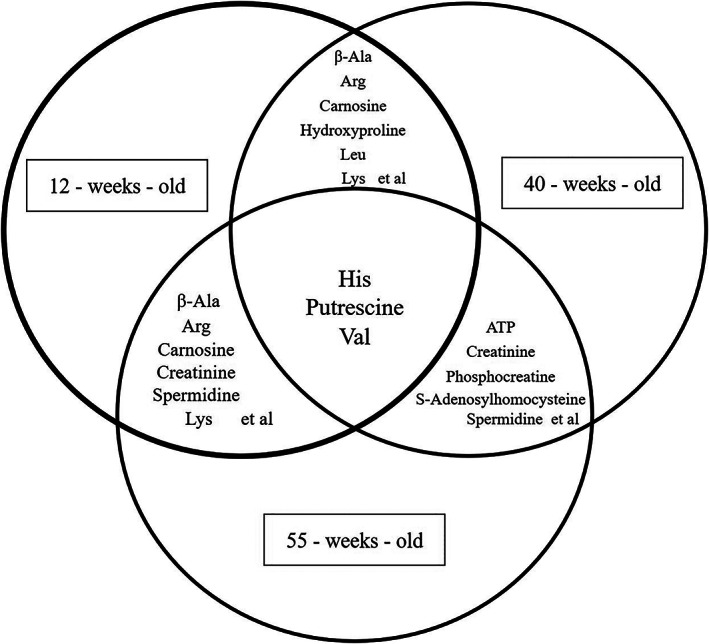
Venn diagram of metabolites that fluctuated in all groups. The fluctuated metabolites that were common among the groups were Val, putrescine and His. ATP, adenosine triphosphate.

Considerable changes in metabolites were observed in the PCA and heat map. The PCA showed that the metabolites contributing to the first principal component could be clearly distinguished at each age, and hierarchical cluster analysis also showed changes in metabolite profiles (Fig. 2). Furthermore, metabolic fluctuations were apparent between young and aged mice, between 12‐ and 40‐weeks‐old, and between 40‐ and 55‐weeks‐old. Among the 116 water‐soluble metabolites associated with the pathway of central energy metabolism in EDL, changes were observed in 19 metabolites between 12‐ and 40‐weesk‐old (Table S1), in 40 metabolites between 40‐ and 55‐weeks‐old (Table S2), and in 57 metabolites between 12‐ and 55‐weeks‐old (Table S3). All the metabolites that fluctuated between 12‐ and 40‐weeks‐old decreased. An increase in the concentration of 16 metabolites and a decrease in the concentration of 24 metabolites were observed between 40‐ and 55‐weeks‐old. An increase in the concentration of 13 metabolites and a decrease in the concentration of 44 metabolites were observed between 12‐ and 55‐weeks‐old. The fluctuated metabolites that were common among the groups were Val, putrescine and His (Fig. 3).

#### Quantitative analysis of metabolites

**Figure 4:**
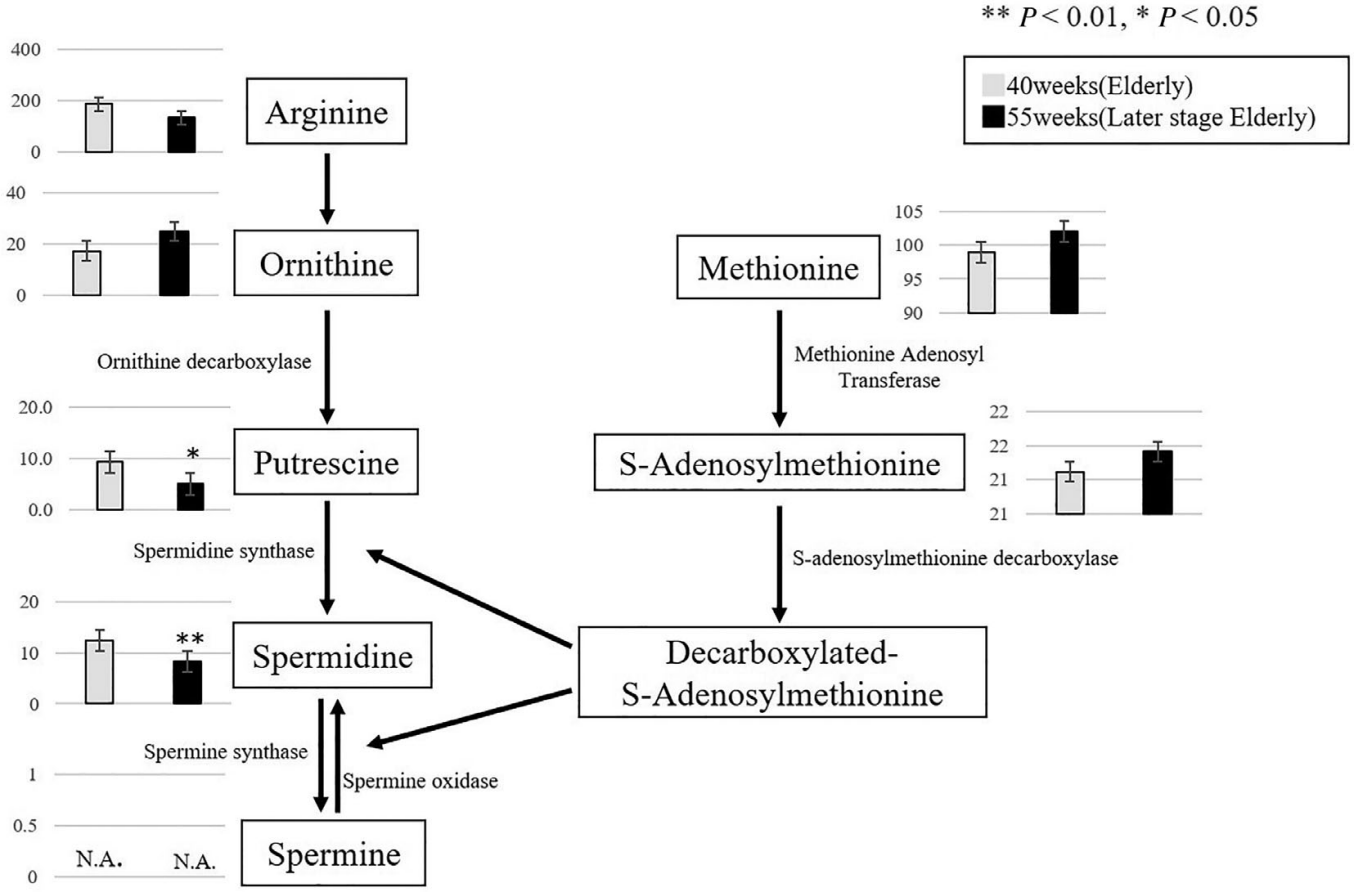
Metabolic changes related to polyamine metabolism between 40‐ and 55‐week‐old senescence‐accelerated mouse‐prone 8 (SAMP8) mice. (**P < 0.01, *P < 0.05).

**Figure 5:**
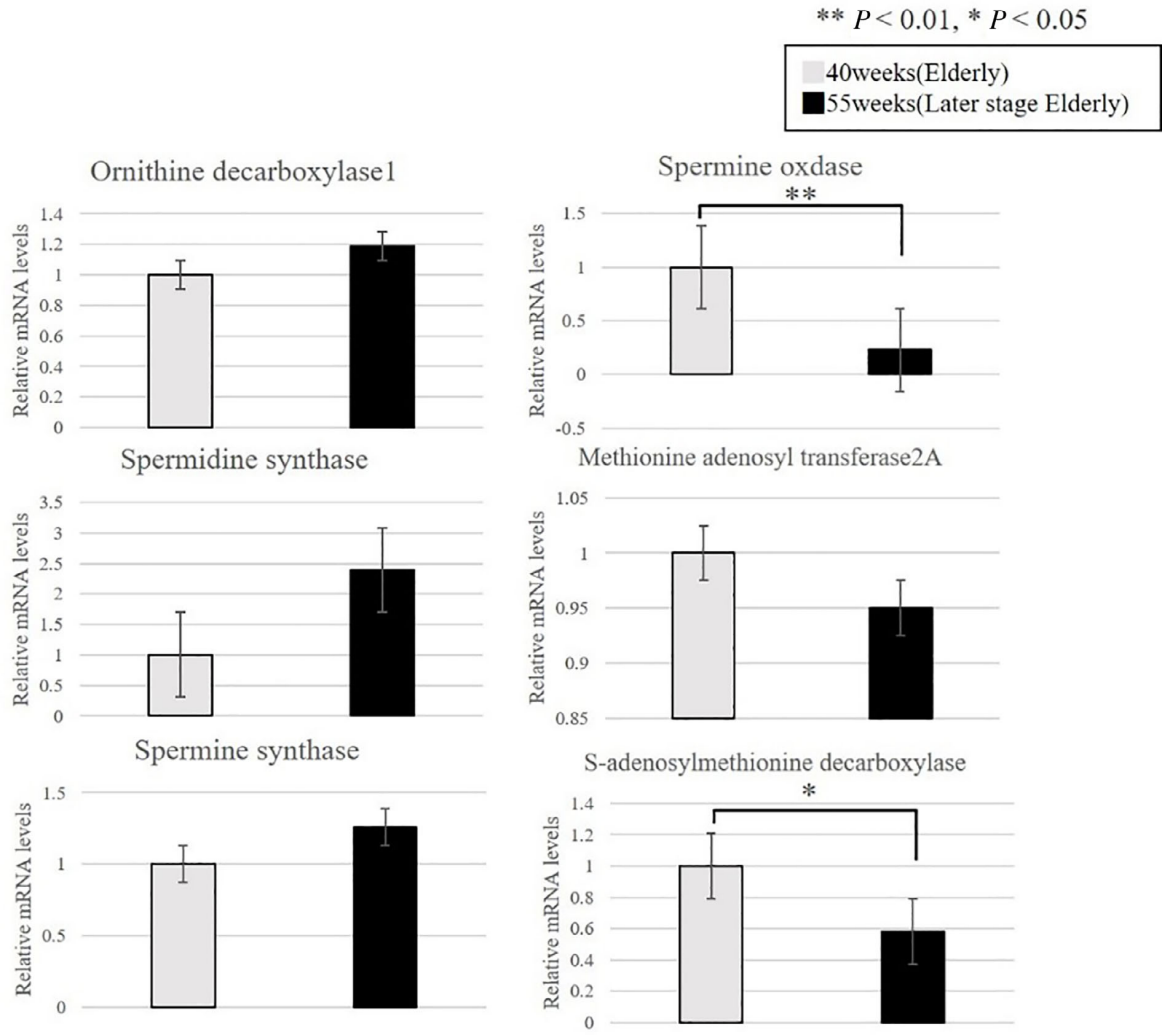
Gene expression changes in polyamine metabolism between 40‐ and 55‐week‐old senescence‐accelerated mouse‐prone 8 (SAMP8) mice. Gray bars: 40‐week‐old SAMP8 mice, black bars: 55‐week‐old SAMP8 mice (**P < 0.01).

We confirmed that the expression of the following enzymes in the polyamine pathway fluctuated with aging: putrescine and spermidine levels decreased in 55‐week‐old mice compared with those in 40‐week‐old mice (Fig. 4). We observed a significant decrease in the expression of the spermine oxidase gene (*Smox*) and S‐adenosylmethionine decarboxylase gene (*Amd2*) in 55‐week‐old mice compared with that in 40‐week‐old mice (Fig. 5).

## Discussion

Metabolites are generated by proteins that are produced per the central dogma biologically. As metabolites occasionally suppress transcriptional expression and protein functions, we considered that investigating metabolites might lay a foundation to elucidate the mechanism of aging in the EDL. The EDL is involved in the flexion and inner movement of the ankle joint, and is an important muscle for walking. The EDL has been studied in aging‐related research,^9^ and that of SAMP8 is mainly composed of type II fiber.^5^ Derave *et al*. observed the decreasing of maximum tetanic force, contraction speed and phosphocreatine content in the EDL of SAMP8 mice.^5^ Phosphocreatine is working as the fastest source of energy during resynthesis of adenosine triphosphate by aging. Although there was no significant change in the muscle mass and its contractile characteristics, a substantial decrement of muscle weakness was observed.

Dynpenia is the age‐associated gradual loss of muscle strength. Dynapenia also encompasses sarcopenia, which is defined as the progressive loss of skeletal muscle mass and strength due to physical disability and poor quality of life. Interestingly, even if the skeletal muscle mass is unperturbed and only the muscle weakness is diminished, the overall quality of the skeletal muscle is critically affected. This leads to a decline in body function, a decrease of the essential amino acids is detected in the blood stream and shortens the survival time.^10^, ^11^ Furthermore, we used EDL in the present study, because sarcopenia is selectively characterized by atrophy of fast‐twitch muscle.

The changes observed due to aging in SAMP8 mice might differ from those in normal human aging. However, SAMP8 mice have a short lifespan, and they have been used in many muscle aging‐related studies.^4^ Moreover, they have a sarcopenia phenotype.^4^ We confirmed the occurrence of chronological changes of aging in the masseter muscle of SAMP8 mice, and we presume that SAMP8 will be used in future studies on aging.^6^

The EDL in SAMP8 mice showed loss of muscle strength and decreased expression of creatine and phosphocreatine, which are involved in muscle movement.^5^ Whereas, the changes in muscle wet weight, cross‐sectional area of muscle fiber and the proportion of MyHC isoform with aging were lower than those in the soleus muscles.^5^ The quantification of MyHC mRNA also showed a tendency of increase in the expression of *MyHC‐1* and *MyHC‐2x/d* from 12‐ to 40‐weeks‐old, and decrease in the expression of *MyHC‐2a* and *MyHC‐2b*; however, there was no significant difference. In contrast, the expression of *MyHC‐1* increased significantly from 12‐ to 55‐week‐old. It has been reported that the isoform of MyHC changes in the fast and slow muscle fibers due to aging.^12^ The significant decrease in myHC‐2 expression at the mRNA level could not be confirmed; however, these phenomena might have occurred partially, leading to an increase in expression of *MyHC‐1*. It has been suggested that with aging, the EDL of SAMP8 mice might undergo molecular biological changes than morphological changes.

Several studies have observed aging‐related morphological and molecular biological changes in the muscles of the lower limbs of aged animal models. The following findings related to molecular biological changes have been reported: (i) muscular atrophy in mouse models in which the transcription factor FOXO1 is overexpressed^13^; (ii) an improvement in motor function in a mouse model in which the transcriptional coactivator, peroxisome proliferator‐activated receptor‐γ coactivator‐1α, is overexpressed when the branched‐chain amino acid metabolic pathway is activated^14^; and (iii) metabolome analysis of the gastrocnemius muscle of a senile mouse model showed a decrease in glucose metabolism and metabolites from the polyamine metabolic pathway, as well as increases in nucleic acids, protein syntheses and neurotransmitters.^15^ In recent years, amino acids have garnered attention, and were divided into nutritional, sensory and biological regulatory function, and play a role in controlling the function of skeletal muscles.^16^ It is considered that the search for amino acids that fluctuate especially with aging is indispensable among metabolites.

Here, carnosine, β‐ala, hydroxyproline, Lys, Leu and Arg were particularly noteworthy among metabolites that fluctuated in absolute quantitation between 12‐ and 40‐week‐old mice. A decrease in carnosine level was also observed in factor loading of the primary principal component, which had the highest contribution rate in the PCA. Carnosine is a dipeptide comprising β‐Ala and His, and it is abundantly present in the muscles and nervous tissues.^17^ It is characterized by its anti‐oxidant and anti‐fatigue effects,^18^ and is one of the nine metabolites that decrease in abundance with aging.^19^ Carnosine is a metabolite that affects the aging of the EDL. β‐Ala is involved in the aging of muscles,^20^ the brain and nerves.^21^ In addition, it has been found that consuming β‐Ala can increase carnosine level by 20–80%.^20^ Here, β‐Ala decreased with His from 12‐ to 40‐weeks‐old and from 12‐ to 55‐weeks‐old. Whereas, the level of only His decreased between 40‐ and 55‐week‐old. Thus, β‐Ala rather than His affects the concentration of carnosine. A decrease in the β‐Ala level might have an effect on the reduced expression of carnosine in the EDL, as well as muscular function. Hydroxyproline is the main component of collagen proteins, in which proline residues are hydroxysyled, and is biosynthesized by protein degradation. It is inferred that the significant decrease in hydroxyproline might decrease the degradation of collagen and the synthesis of proteins. The weight of the soleus muscles in SAMP8 mice that were provided Lys (which is present in muscles especially)‐containing feed recovered to a level equivalent to that of the control group, SAM‐R1.^22^ The expression of LC‐III, which is an indicator of the protein degradation mechanism, autophagy activity, is also suppressed in SAMP8 mice. Supplementation with Lys might cause similar changes in not only the soleus muscles, but also the EDL, because it was also significantly decreased in the EDL in the present study. Leu and Val of branched‐chain amino acids, which are commonly known amino acids that stimulate protein synthesis, and Arg affect the aging of the EDL. Leu activates mechanistic target of rapamycin complex 1, which controls intracellular antholytic reactions, and functions as a stimulating factor in the synthesis of muscle proteins.^16^ In addition, Arg is specifically detected by CASTOR1 and activates mechanistic target of rapamycin complex 1.^23^ Both were involved in protein synthesis, and the decrease could be due to aging of the EDL in SAMP8 mice. Furthermore, Val, an amino acid secreted by skeletal muscle, especially during exercise, acts on other tissues to increase energy expenditure. In the present study, there was a significant decrease in Val level in 40‐ and 55‐week‐old mice compared with that in 12‐week‐old mice. It has been reported that 3‐hydroxyisobutyric acid, an intermediate metabolite of Val, stimulates the absorption of fatty acids in skeletal muscle and leads to insulin resistance.^16^ The branched‐chain amino acid metabolic pathway might be important from the perspective of aging of the EDL in SAMP8 mice, because a lack of the metabolic pathway is associated with obesity and diabetes.

We confirmed a decrease in glycolytic enzymes, such as fructose 6‐phosphate, glucose 6‐phosphate, glycerol 3‐phosphate and dihydroxyacetate phosphate, between 40‐ and 55‐week‐old mice. Furthermore, the levels of putrescine and spermidine involved in the polyamine pathway, and spermine oxidase and S‐adenosylmethionine involved in the expression of metabolites of the polyamine pathway decreased in the EDL of SAMP8 mice. Spermidine is an autophagy‐related autoinducer, and its concentration decreased in aged mice.^24^

The results of the present study in the EDL are similar to those of Uchitomi *et al*.^15^ As the levels of enzymes involved in the polyamine pathway also decreased, it was thought that the enzymes might be involved in changes due to aging in cell proliferation, protein synthesis and nucleic acid synthesis. Putrescine is a metabolite produced by ornithine decarboxylase, which is also inferred to be involved in the reduction of spermine. It has been reported that DNA methylation is caused by *S‐*adenosylmethionine in the polyamine metabolic pathway.^25^ The expression of *S‐*adenosylmethionine might also decrease with a decrease in the level of S‐adenosylmethionine decarboxylase. As abnormal methylation of DNA has also been reported with aging, it might be necessary to consider the effect of methylation of the entire gene by polyamine on the EDL of SAMP8 mice.^26^ The polyamine metabolic pathway is also associated with lipid oxidation β.^27^, ^28^ In the present study, we observed a decrease in the acetyl CoA and malonyl CoA levels. This suggests changes in lipid metabolism, which is an important energy source for skeletal muscle. Furthermore, we observed a decrease in the levels of creatine and phosphocreatine, which are involved in muscular movement.^29^

To prevent sarcopenia and maintain the walking function, it is important to show the metabolites that are influenced by aging in the EDL. We confirmed that there were two metabolic changes related to aging in the extensor digitorum longus muscle of SAMP8 mince. Based on the changes in the aforementioned metabolites, muscular senescence and fatigue in the EDL might increase in old mice compared with those in young mice, indicating molecular biological changes. Ingestion of significantly fluctuating metabolites might lead to the maintenance of muscle function and the prevention of sarcopenia. Furthermore, activation of polyamine metabolism and glycolysis that fluctuate from 40‐ and 55‐weeks‐old might extend the healthy life expectancy in aging societies. Carrying out additional gene expression analyses, such as microarray and bioinformatic analyses, might help understand the aging mechanism of the EDL.

## Disclosure statement

The authors declare no conflict of interest.

## Supporting information


**Table S1** Metabolites with significant differences in absolute quantitative values in capillary electrophoresis–mass spectrometry metabolome analysis between 12‐ and 40‐week‐old senescence‐accelerated mouse‐prone 8 mice. *P*‐values were determined using the Welch *t*‐test.

**Table S2** Metabolites with significant differences in absolute quantitative values in capillary electrophoresis–mass spectrometry metabolome analysis between 40‐ and 55‐week‐old senescence‐accelerated mouse‐prone 8 mice. *P*‐values were determined using the Welch *t*‐test.

**Table S3** Metabolites with significant differences in absolute quantitative values in capillary electrophoresis–mass spectrometry metabolome analysis between 12‐ and 55‐week‐old senescence‐accelerated mouse‐prone 8 mice. *P*‐values were determined using the Welch *t*‐test.

## Data Availability

The data that support the findings of this study are available from the corresponding author upon reasonable request.
